# Newly designed Protein Transduction Domain (PTD)‐mediated BMP‐7 is a potential therapeutic for peritoneal fibrosis

**DOI:** 10.1111/jcmm.15992

**Published:** 2020-10-20

**Authors:** Seonghun Kim, Dong Ho Shin, Bo Young Nam, Hye‐Young Kang, Jimin Park, Meiyan Wu, Nam Hee Kim, Hyun Sil Kim, Jung Tak Park, Seung Hyeok Han, Shin‐Wook Kang, Jong In Yook, Tae‐Hyun Yoo

**Affiliations:** ^1^ Department of Oral Pathology Oral Cancer Research Institute Yonsei University College of Dentistry Seoul Korea; ^2^ Department of Internal Medicine Kangdong Sacred Heart Hospital Hallym University College of Medicine Seoul Korea; ^3^ Severance Biomedical Science Institute Yonsei University College of Medicine Seoul Korea; ^4^ Department of Internal Medicine Brain Korea 21 PLUS Project for Medical Science Yonsei University College of Medicine Seoul Korea; ^5^ Department of Nephrology The First Hospital of Jilin University Changchun China; ^6^ Division of Nephrology Department of Internal Medicine College of Medicine Yonsei University Seoul South Korea; ^7^ MET Life Science Seoul Korea

**Keywords:** and peritoneal fibrosis, protein transduction domain, PTD‐BMP‐7

## Abstract

While the bone morphogenetic protein‐7 (BMP‐7) is a well‐known therapeutic growth factor reverting many fibrotic diseases, including peritoneal fibrosis by peritoneal dialysis (PD), soluble growth factors are largely limited in clinical applications owing to their short half‐life in clinical settings. Recently, we developed a novel drug delivery model using protein transduction domains (PTD) overcoming limitation of soluble recombinant proteins, including bone morphogenetic protein‐7 (BMP‐7). This study aims at evaluating the therapeutic effects of PTD‐BMP‐7 consisted of PTD and full‐length BMP‐7 on epithelial‐mesenchymal transition (EMT)‐related fibrosis. Human peritoneal mesothelial cells (HPMCs) were then treated with TGF‐β1 or TGF‐β1 + PTD‐BMP‐7. Peritoneal dialysis (PD) catheters were inserted into Sprague‐Dawley rats, and these rats were infused intra‐peritoneally with saline, peritoneal dialysis fluid (PDF) or PDF + PTD‐BMP‐7. In vitro, TGF‐β1 treatment significantly increased fibronectin, type I collagen, α‐SMA and Snail expression, while reducing E‐cadherin expression in HPMCs (*P* < .001). PTD‐BMP‐7 treatment ameliorated TGF‐β1‐induced fibronectin, type I collagen, α‐SMA and Snail expression, and restored E‐cadherin expression in HPMCs (*P* < .001). In vivo, the expressions of EMT‐related molecules and the thickness of the sub‐mesothelial layer were significantly increased in the peritoneum of rats treated with PDF, and these changes were significantly abrogated by the intra‐peritoneal administration of PTD‐BMP‐7. PTD‐BMP‐7 treatment significantly inhibited the progression of established PD fibrosis. These findings suggest that PTD‐BMP‐7, as a prodrug of BMP‐7, can be an effective therapeutic agent for peritoneal fibrosis in PD patients.

## 1 INTRODUCTION

Peritoneal dialysis (PD) is a modality of renal replacement therapy using a semipermeable membrane where ultrafiltration and diffusion occur.^1^ As PD duration prolongs, prolonged exposure to bio‐incompatible PD solutions and recurrent peritonitis causes chronic inflammation and subsequent injury to the peritoneal membrane, which undergoes progressive fibrosis and angiogenesis, eventually leading to ultrafiltration loss.^2^

Epithelial‐mesenchymal transition (EMT) of the peritoneal mesothelial cells (PMCs) is a key process of peritoneal fibrosis.^2^ Transforming growth factor‐β1 (TGF‐β1) and bone morphogenetic protein‐7 (BMP‐7) are two key factors in the balance of their biological activities in EMT of PMCs by counter‐regulatory mechanism.^3^, ^4^, ^5^ TGF‐β1, a strong profibrotic cytokine, is known as a master molecule in the structural change and functional deterioration of the peritoneal membrane.^6^ On the other hand, BMP‐7, which is an endogenous anti‐fibrotic protein via the blocking activation of downstream Smad 2/3 cascades, restores E‐cadherin expression to normal levels through Smad 1/5/8 up‐regulation, suggesting repair of severely damaged PMCs.^7^, ^8^, ^9^ In fact, some studies showed that recombinant BMP‐7 (rBMP‐7) or adenovirus‐mediated expression of BMP‐7 reversed the TGF‐β1‐induced EMT and peritoneal fibrosis in in vitro or in vivo models.^3^, ^4^, ^5^ Although rBMP‐7 presents as a promising therapeutic for many TGF‐β1‐dependent fibrotic diseases, there are several critical limitations due to soluble nature of recombinant proteins. For example, unlike in vitro culture dish, intra‐peritoneal administration of soluble and bioactive rBMP‐7 in vivo inevitably leads to their initial burst release and short half‐life because of rapid clearance by body fluid and proteolytic degradation.^10^, ^11^, ^12^ Because of these limitations, excess dosages of rBMP‐7 are often administered to achieve clinical effectiveness. Therefore, a high dosage of rBMP‐7, during a long‐term regeneration period (often several weeks), inevitably increases the possibility of spreading to nearby tissues or blood circulation with many adverse effects, while remaining cost‐ineffective. While adenovirus‐mediated BMP‐7 can provide long‐term release, viral transduction for clinical therapeutics is largely limited because of safety concerns.

Recently, it has been found that protein transduction domain (PTD) peptides deliver proteins into the cell across the plasma membrane as a part of the physiologic process.^13^ In terms of therapeutic benefits without raising safety concerns regarding gene delivery, PTD is widely investigated as carrier for therapeutic macromolecules.^14^ The PTD‐fusion polypeptide is rapidly internalized through lipid raft‐dependent macropinocytosis after the initial ionic contact with the cell membrane independent of specific receptors.^15^ Interestingly, the denatured polypeptides delivered into the cells refold into active form.^16^, ^17^ Unfortunately, the mechanism detailing the intracellular processing of the delivered protein is not yet to be fully explained.^16^, ^17^, ^18^ We have previously reported that PTD‐mediated transduction of BMP provides a novel drug delivery model overcoming limitations of current soluble recombinant proteins.^12^ Therefore, this study was undertaken to evaluate the effect of PTD‐BMP‐7 on TGF‐β1‐induced EMT in cultured human peritoneal mesothelial cells (HPMCs). Furthermore, the effects of PTD‐BMP‐7 on EMT and peritoneal fibrosis were investigated in peritoneal dialysis fluid (PDF)‐induced peritoneal fibrosis animal models.

## 2 MATERIALS AND METHODS

### 2.1 Ethnic statement

The study was approved by the Committee for the Care and Use of Laboratory Animals at Yonsei University College of Medicine (Seoul, Republic Korea), according to the Principles of Laboratory Animal Care (NIH Publication no. 85‐23, revised 1985).

### 2.2 Bacterial expression, production of PTD‐BMP‐7, and isolation of HPMCs

The bacterial expression cassette for the PTD‐fusion BMP‐7 polypeptide and the purification procedures have been described previously.^12^ HPMCs were isolated in accordance with the process stated by Stylianou et al.^19^

### 2.3 Transduction of PTD‐BMP‐7 into HPMCs

PTD‐BMP‐7 was directly introduced into the HPMC culture medium for 24 hours. Using immunofluorescence analysis, PTD‐BMP‐7 was detected via the antibodies against the BMP‐7 (ab29569, Abcam). Of note, 4′,6‐diamidino‐2‐phenylindole (DAPI, Invitrogen) to counterstain nuclei was used. Additionally, HPMCs were grown in 10% foetal bovine serum (FBS)‐free M199 media (Sigma‐Aldrich Corp). FBS‐free M199 media + 4.25% PDF (Physioneal; Baxter International, Deerfield, IL, USA). HPMCs were also treated with different doses of PTD‐BMP‐7 for 2 hours. The soluble and insoluble fractions of lysate were obtained from Triton X‐100 lysis buffer. Xpress and BMP‐7 protein expressions of insoluble fractions were analysed using Western blot analysis. To determine adequate processing of PTD‐BMP‐7 and secretion of BMP‐7 in HPMCs, PTD‐BMP‐7 was transduced into the HPMCs for 2 hours, followed by sterile phosphate‐buffered saline (PBS) washing and refreshment of FBS‐free M199 media containing Noggin (SRP4675; Sigma‐Aldrich Corp.), an endogenous inhibitor which binds competitively BMP‐7 receptor on the cell membrane. After 4 hours of incubation with PTD‐BMP‐7, the culture media was harvested and subjected to BMP‐7 ELISA assay kit (DBP700; R&D Systems) according to the manufacturer's protocol to evaluate secreted BMP‐7.

### 2.4 In vitro experiments

3‐(4,5 dimethylthiazol‐2‐yl)‐2,5‐diphenyltetrazolium bromide (MTT) cell viability assay kit (Thermo Fisher Scientific) was performed to determine the PTD‐BMP‐7 dose used in the experiments.^20^ Subconfluent HPMCs were FBS‐restricted for 24 hours after which the media was replaced into 1% FBS media for the control group and the same media with TGF‐β1 (2 ng/mL) (R&D Systems) for the TGF‐β1 group. Both groups were treated with PTD‐BMP‐7 (100 ng/mL). Preliminary experiments determined the doses of TGF‐β1 and PTD‐BMP‐7 in this study. Cells were harvested 72 hours after treatment with each stimulus.

### 2.5 Animal experiments

Peritoneal access ports were implanted in male Sprague‐Dawley rats weighing 200‐250 g, and 2 mL of saline containing 1 IU/mL of heparin was injected intra‐peritoneally until the wound healed. First, control rats (n = 6) were instilled with a daily (once per day) 20 mL dose of saline. The remaining rats (n = 12) were injected daily with 20 mL of 4.25% PDF for 4 weeks with or without PTD‐BMP‐7 (50 μg/kg, weekly). The PTD‐BMP‐7 was mixed with PDF prior to intra‐peritoneal administration (PDF + PTD‐BMP‐7). Of note, to evaluate the effects of PTD‐BMP‐7 on the prevention of peritoneal fibrosis based on the dosage of PTD‐BMP‐7, we performed the same procedure in the 18 male Sprague‐Dawley rats. Among them, each three rats were instilled with 4.25% PDF + saline (20 mL/d), or different doses of PTD‐BMP‐7 (0.05, 0.5, 5, 50 and 500 μg/kg/wk) for 4 weeks. Next, to evaluate the effects of PTD‐BMP‐7 on the established PDF‐induced peritoneal fibrosis, we performed the same procedure in 30 male Sprague‐Dawley rats. Each of six rats was instilled with a daily (once per day) 20 mL dose of saline with or without PTD‐BMP‐7 (50 μg/kg, weekly), and the remaining 18 rats were injected with 20 mL of PDF daily for 6 weeks. Among those, six rats were injected with a mixture of PTD‐BMP‐7 (50 μg/kg, weekly) and PDF for 6 weeks (PDF + 1‐6 weeks of PTD‐BMP‐7), while 6 rats were treated with PTD‐BMP‐7 (50 μg/kg, weekly) after 2 weeks of PDF treatment (PDF + 3‐6 weeks of PTD‐BMP‐7). Lastly, to compare the effectiveness of PTD‐BMP‐7 to those of rBMP‐7, we also performed the same procedure in 36 male Sprague‐Dawley rats. Among them, each six rats were instilled with saline (20 mL/d), saline (20 mL/d) + PTD‐BMP‐7 (50 μg/kg, weekly) and saline (20 mL/d) + rBMP‐7 (50 μg/kg, weekly). The remaining 18 rats were injected with 20 mL of PDF daily for 4 weeks. Among those, six rats were injected with rBMP‐7 (50 μg/kg, weekly) + PDF for 4 weeks, while 6 rats were injected with PTD‐BMP‐7 (50 μg/kg, weekly) + PDF for 4 weeks.

### 2.6 Total RNA extraction and reverse transcription

The RNA extraction method from HPMCs was described in a previous study.^21^ Extracted RNA was precipitated by adding 400 µl of isopropanol and centrifuged at 12 000 *g* for 30 minutes at 4°C. The RNA pellet was washed with 70% ethanol, air‐dried for 2 minutes and dissolved in sterile DEPC‐treated distilled water. A Boehringer Mannheim cDNA synthesis kit (Boehringer Mannheim GmbH) was used to obtain first‐strand cDNA. Reverse transcription was conducted using 2 µg of total RNA extracts with 10 µmol/L random hexanuclotide primers, 1 mmol/L dNTPs, 8 mmol/L MgCl_2_, 30 mmol/L KCl, 50 mmol/L Tris‐HCl at pH 8.5, 0.2 mmol/L dithiothreithol, 25 U RNAse inhibitor and 40 U AMV reverse transcriptase. The mixture was incubated for 10 minutes at 30°C, and then for 1 hour at 42°C, followed by incubation for 5 minutes at 99°C for the inactivation of the enzyme.

### 2.7 Quantitative real‐time polymerase chain reaction (qRT‐PCR)

The primer sequences used in this study were described in Table S1. The RNAs used for amplification were 25 ng per reaction tube. Using the ABI PRISM 7700 Sequence Detection System (Applied Biosystems), a total volume of 20 µL mixture in each well was used containing 10 µL of SYBR Green PCR Master Mix (Applied Biosystems), 5 µL of cDNA, and 5 pmol sense and antisense primers. The PCR conditions were as follows: 35 cycles of denaturation for 30 minutes at 94.5°C, annealing for 30 seconds at 60°C and extension for 1 minutes at 72°C. Initial heating for 9 minutes at 95°C and final extension for 7 minutes at 72°C were performed for all PCR reactions. After real‐time PCR, the temperature was increased from 60 to 95°C at a rate of 2°C/min to construct a melting curve. The cDNA content of each specimen was determined using a comparative C_T_ method with 2^–ΔΔCT^. The results were given as the relative expression normalized to the expression of 18S rRNA and expressed in arbitrary unit.

### 2.8 Western blot analysis

Western blot analysis of harvested cultured cells and the mouse peritoneum were conducted. Harvested cultured cells and the mouse peritoneum were lysed in sodium dodecyl sulphate (SDS) sample buffer [2% SDS, 10 mmol/L Tris‐HCl at pH 6.8, 10% (vol/vol) glycerol]. Lysates were centrifuged at 10 000 *g* for 10 minutes at 4°C, and the supernatant was stored at −70°C. Protein concentrations were determined using the Bio‐Rad kit (Bio‐Rad Laboratories, Inc). Laemmli sample buffer was added to aliquots of 50 µg of the protein extracts, which were heated for 5 minutes at 100°C and electrophoresed in acrylamide denaturating SDS‐polyacrylamide gel. A Hybond‐ECL membrane was used to transfer protein using a Hoeffer semidry blotting apparatus (Hoeffer Instruments). After the protein was transferred to the membrane, it was incubated in blocking buffer A (1X PBS, 0.1% Tween‐20 and 5% nonfat milk) for 1 hour at room temperature, and then incubated overnight at 4°C in a 1:500 dilution of polyclonal antibodies to Xpress (46‐0528; Invitrogen), BMP‐7 (ab84684; Abcam), fibronectin (A0245; Dako, Glostrup, Denmark), type I collagen (1310‐01; SothernBiotech), α‐SMA (ab5694; Abcam), E‐cadherin (610181; BD Bioscience), Snail (ab85936; Abcam), phospho‐Smad 2/3 (p‐Smad 2/3, 8828S; Cell Signaling Technology Inc), Smad 2/3 (3102S; Cell Signaling Technology Inc) or β‐actin (A5441; Sigma Chemical Co.). The membranes were washed thrice for 10 minutes in 1 × PBS with 0.1% Tween‐20 and incubated in buffer A containing a 1:1000 dilution of horseradish peroxidase‐linked goat anti‐rabbit or anti‐mouse IgG (Santa Cruz Biotechnology, Inc). The washing process was repeated thrice, and the membranes were developed with a chemiluminescent agent (ECL; Amersham Life Science, Inc). TINA image software (Raytest, Straubenhardt, Germany) was used to measure the band densities, and the changes in the optical densities of bands from the treated groups relative to control cells or tissues were used for analysis.

### 2.9 Immunohistochemical staining and Masson's trichrome staining

The peritoneum samples were fixed in 10% neutral‐buffered formalin, processed in the standard manner, and 5 µm‐thick sections of paraffin‐embedded tissues were utilized for immunohistochemical staining. Slide was deparaffinized, hydrated in ethyl alcohol and washed in tap water. Antigen retrieval was performed in 10 mmol/L sodium citrate buffer for 20 minutes using a Black & Decker vegetable steamer. Slides were blocked with 10% donkey serum for 30 minutes at room temperature and then washed using PBS. Primary antibodies for fibronectin, E‐cadherin, α‐SMA and Snail were diluted 1:100 with 2% casein in bovine serum albumin, added to the slides and then incubated overnight at 4°C. After washing, a secondary antibody was added for 20 minutes, and the slides were washed and incubated with a tertiary PAP complex for 20 minutes. Diaminobenzidine was added for 2 minutes and the slides were counterstained with haematoxylin. A semi‐quantitative score of staining intensity was determined by examining at least 5 fields of the peritoneum in each section under ×400 magnification and with digital image analysis (MetaMorph version 4.6r5, Universal Imaging Corp.). For Masson's trichrome staining, paraffin‐embedded tissues processed in 5 µm‐thick sections were deparaffinized, rehydrated in ethyl alcohol, washed in tap water and re‐fixed in Bouin's solution for 1 hour at 56°C. After rinsing in running tap water for 10 minutes and staining with Weigert's iron haematoxylin working solution for 10 minutes, the slides were stained with Biebrich scarlet‐acid fuchsin solution for 15 minutes and washed in tap water. The sections were differentiated in phosphomolybdic‐phosphotungstic acid solution for 15 minutes, transferred to aniline blue solution and stained for 10 minutes. After rinsing briefly in tap water, the slides were reacted with 1% acetic acid solution for 5 minutes. The thickness of the peritoneum, which was defined as the tissue between the mesothelial surface and the underlying muscle or parenchyma, was assessed as previously described.^22^

### 2.10 Intravital fluorescence imaging

Female 5 week‐age Balb/C nude mice for in vivo (n = 2/group) or ex vivo (n = 3) fluorescence imaging assay were fed with alfalfa‐free diet after adaptation period. PTD‐BMP (10 μg/100 μL) and rBMP (10 μg/100 μL) which was labelled with ICG were injected intra‐peritoneally. In vivo intravital fluorescence imaging was taken immediately before and after PTD‐BMP or rBMP injection, and measured by using VISQUE^®^ InVivo Elite (Vieworks, Anyang, South Korea) under inhalation anaesthesia by Isoflurane after 1, 2, 6, 24, 30, 48, 54, 72, 78 and 168 hours later from initial injection. Ex vivo intravital fluorescence imaging of the mice skin and peritoneum was performed after 6 hours PTD‐BMP injection by using VISQUE^®^ InVivo Smart (Vieworks). All imaging data were analysed by CleVue™ software (Vieworks).

### 2.11 Statistical analysis

Statistical analyses were conducted by the SPSS software Ver. 21.0 for Windows (SPSS, Inc). Data are expressed as mean ± standard errors of the mean (SEM). A one‐way ANOVA with the Bonferonni multiple comparison test was used to analyse data. *P* < .05 was considered to be statistically significant.

## 3 RESULTS

### 3.1 Transduction of PTD‐BMP‐7 and secretion of BMP‐7 in HPMCs

**Figure 1:**
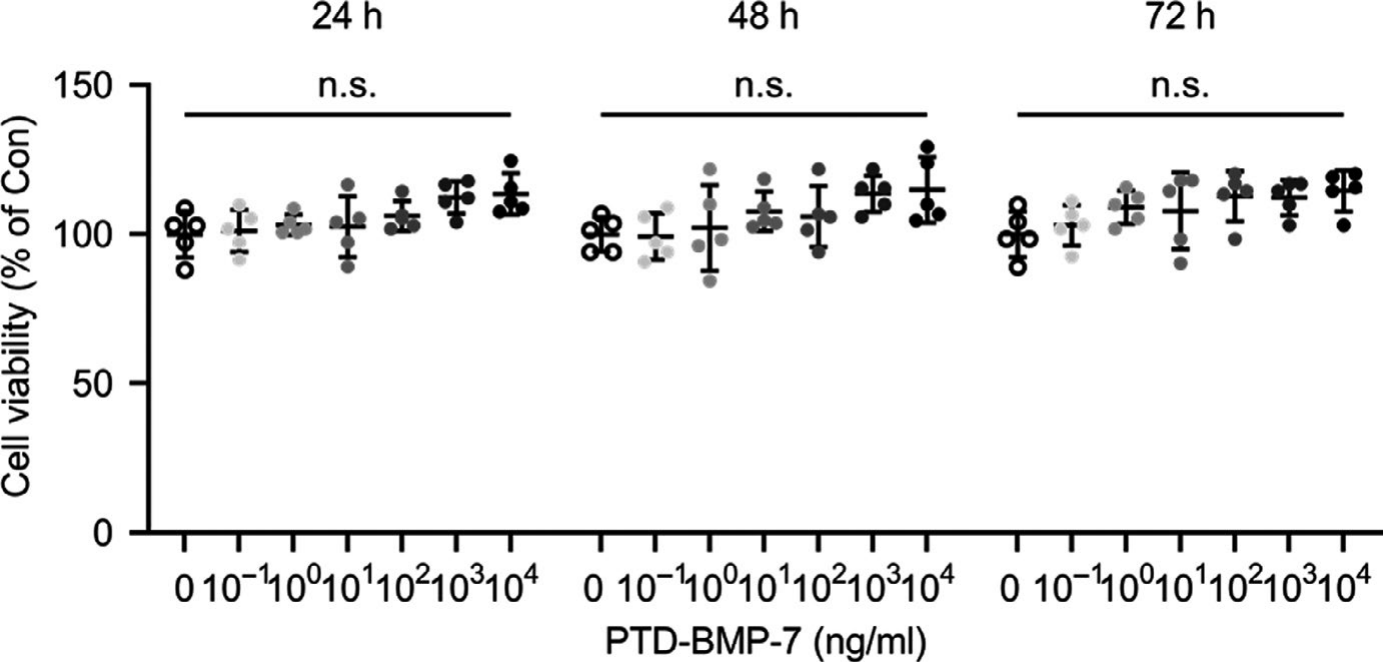
Cytotoxicity of PTD‐BMP‐7 in HPMCs. A, HPMCs were incubated for 72 h with TRP2. Cell viability was maintained at up to 10^4^ ng/mL. ns, not significant vs Con. Data were presented as mean ± SE. Con, control

**Figure 2:**
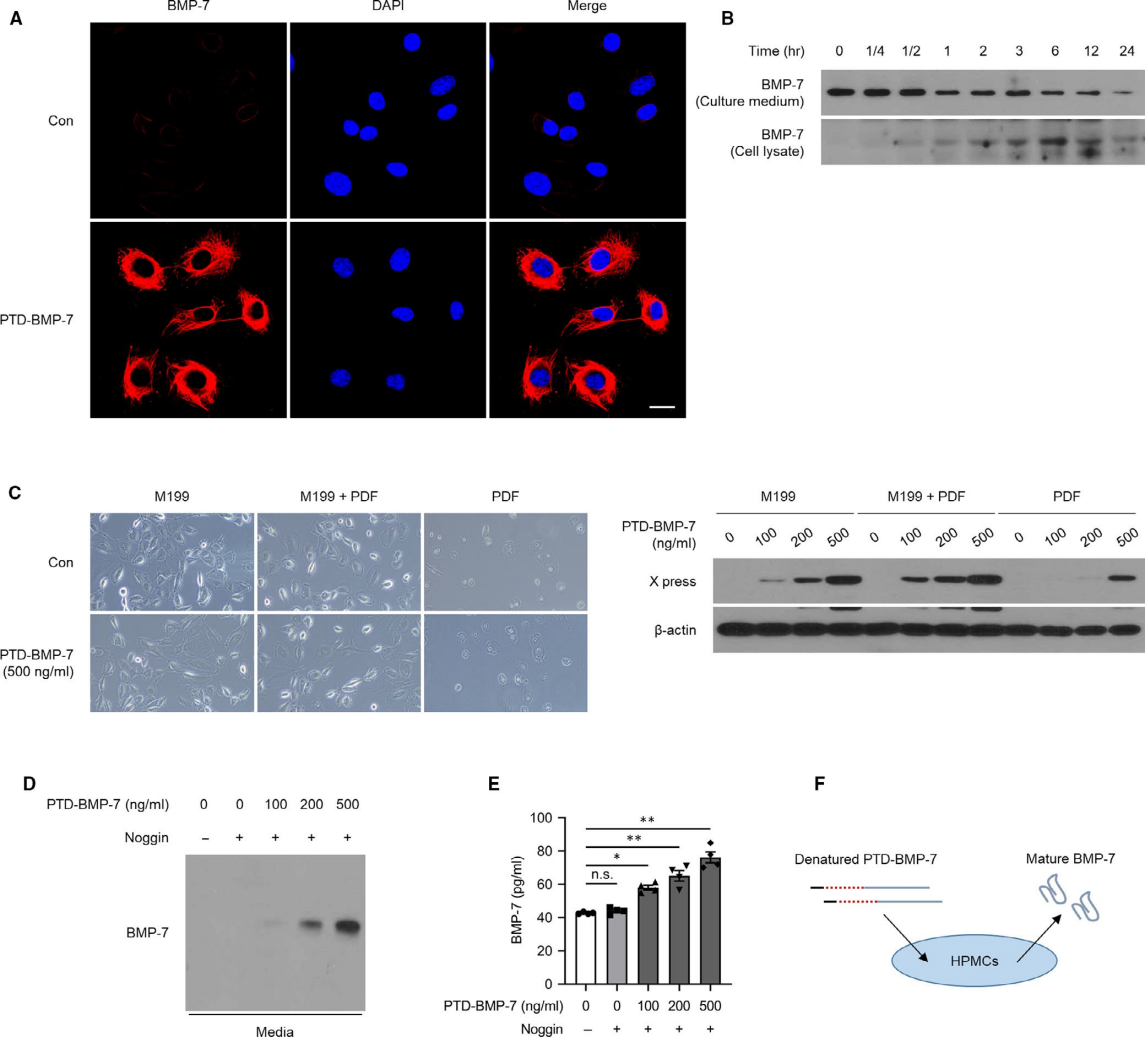
Transduction of PTD‐BMP‐7 and secretion of BMP‐7 in HPMCs. A, HPMCs were transduced with 500 ng/mL of PTD‐BMP‐7 and transduction of PTD‐BMP‐7 (red) was determined via confocal laser microscopy (×400). Nuclei are stained blue using 4′,6‐diamidino‐2‐phenylindole (DAPI). B, Transduction of PTD‐BMP‐7 into cells in a time‐dependent manner. Total protein containing insoluble fraction and media were subjected to Western blot analysis to detect transduction of PTD‐BMP‐7 in HPMCs. C, HPMCs were treated with different doses of PTD‐BMP‐7 in FBS‐free M199 media, FBS‐free M199 media + PDF and PDF. The protein expression of the X press in the cell lysates increased in a PTD‐BMP‐7‐dose‐dependent manner. D, E, Secreted BMP‐7 protein in FBS‐free M199 media was successfully detected in a PTD‐BMP‐7‐dose‐dependent manner (E; Western blot, F; ELISA). *P < .01 vs PTD‐BMP‐7 0/Noggin +; **P < .001 vs PTD‐BMP‐7 0/Noggin +. F, Schematic diagram for intracellular transduction of PTD‐BMP‐7 and secretion of mature BMP‐7. Representative data from 4 individual experiments are shown. Data were presented as mean ± SE. Con, control; HPMCs, human peritoneal mesothelial cells; FBS, 10% foetal bovine serum; PDF, 4.25% peritoneal dialysis fluid

HPMCs remained viable up to 10^4^ μg/mL of PTD‐BMP‐7 and incubated for 24, 48 and 72 hours, which suggested that PTD‐BMP‐7 did not exhibit cellular toxicity at that dose in HPMCs (Figure 1A). The immunofluorescence and Western blot analysis study demonstrated the presence of PTD‐BMP‐7 in HPMCs (Figure 2A,B), indicating the successful delivery of PTD‐BMP‐7 into the cells. Western blot analysis also revealed that PTD‐BMP‐7 was successfully delivered into the cells grown in FBS‐free M199 culture media, M199 media + PDF and only PDF in a dose‐dependent manner (Figure 2C). Next, we examined whether PTD‐BMP‐7 was adequately processed and BMP‐7 was secreted in HPMCs. When we specifically added Noggin (4 μg/mL) as a BMP‐7 receptor antagonist, the secreted BMP‐7 protein in the FBS‐free M199 media was successfully detected based on the dosage of PTD‐BMP‐7 (Figure 2D,E). These results indicated that the denatured, insoluble form of PTD‐BMP‐7 was successfully transduced into HPMCs and that the cells secreted soluble mature BMP‐7 via intracellular processing (Figure 2F).

### 3.2 Effects of PTD‐BMP‐7 on TGF‐β1‐induced EMT in HPMCs

**Figure 3:**
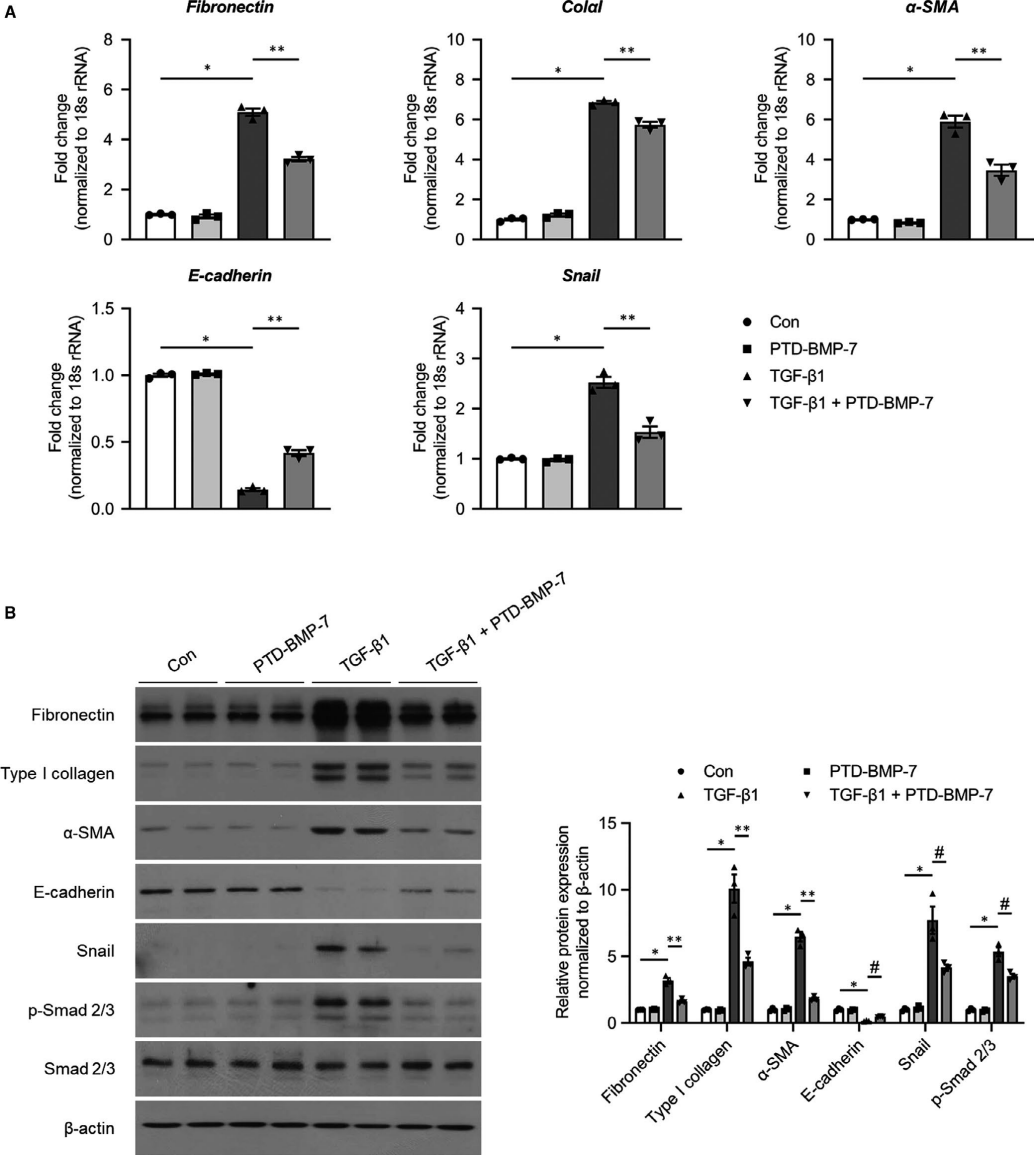
Effects of PTD‐BMP‐7 on EMT‐related fibrosis in TGF‐β1‐treated HPMCs. HPMCs were incubated for 72 h with FBS‐free M199 media (Con), PTD‐BMP‐7 (100 ng/mL), TGF‐β1 (2 ng/mL) and TGF‐β1 + PTD‐BMP‐7 (100 ng/mL). A, TGF‐β1 significantly induced fibronectin, ColαI, α‐SMA, and Snail mRNA expression and significantly reduced E‐cadherin mRNA level in HPMCs when compared to control cells. These changes were significantly attenuated by PTD‐BMP‐7. *P < .001 vs Con; **P < .001 vs TGF‐β1. B, Protein expressions of fibronectin, type I collagen, α‐SMA, E‐cadherin and Snail showed similar expression patterns to the mRNA expression in TGF‐β1‐treated HPMCs. Phospho‐Smad 2/3 protein expression, which induced EMT, was also significantly higher in TGF‐β1‐treated HPMCs. However, the changes in TGF‐β1‐treated HPMCs were significantly abrogated by PTD‐BMP‐7. *P < .001 vs Con; *P *< .001 vs TGF‐β1; #*P *< .01 vs. TGF‐β1. Representative data from 3 individual experiments are shown. Data were presented as mean ± SE. Con, control; HPMCs, human peritoneal mesothelial cells; FBS, 10% foetal bovine serum; PDF, 4.25% peritoneal dialysis fluid

To evaluate the in vitro effects of PTD‐BMP‐7 on EMT, HPMCs were incubated for 72 hours with FBS‐free M199 media (control), PTD‐BMP‐7 (100 ng/mL), TGF‐β1 (2 ng/mL) and TGF‐β1 + PTD‐BMP‐7. The mRNA expression of E‐cadherin was significantly lower (*P* < .001), whereas the mRNA expressions of fibronectin, collagen type I α1 (ColαI), α‐SMA and Snail were significantly higher in TGF‐β1‐treated HPMCs compared to control cells (*P* < .001). The changes in HPMCs exposed to TGF‐β1 were significantly abrogated by PTD‐BMP‐7 treatment (Figure 3A). The protein expressions of E‐cadherin, fibronectin, type I collagen, α‐SMA and Snail showed a similar pattern to the mRNA expressions in TGF‐β1‐treated HPMCs (Figure 3B). Phospho‐Smad 2/3 (p‐Smad 2/3) protein expression, as a downstream signalling molecule of TGF‐β1, was significantly higher in TGF‐β1‐treated HPMCs compared to control cells; however, PTD‐BMP‐7 treatment reduced TGF‐β1‐induced Smad 2/3 phosphorylation in HPMCs (Figure 3B).

### 3.3 Effects of PTD‐BMP‐7 on the development of peritoneal EMT‐related fibrosis

**Figure 4:**
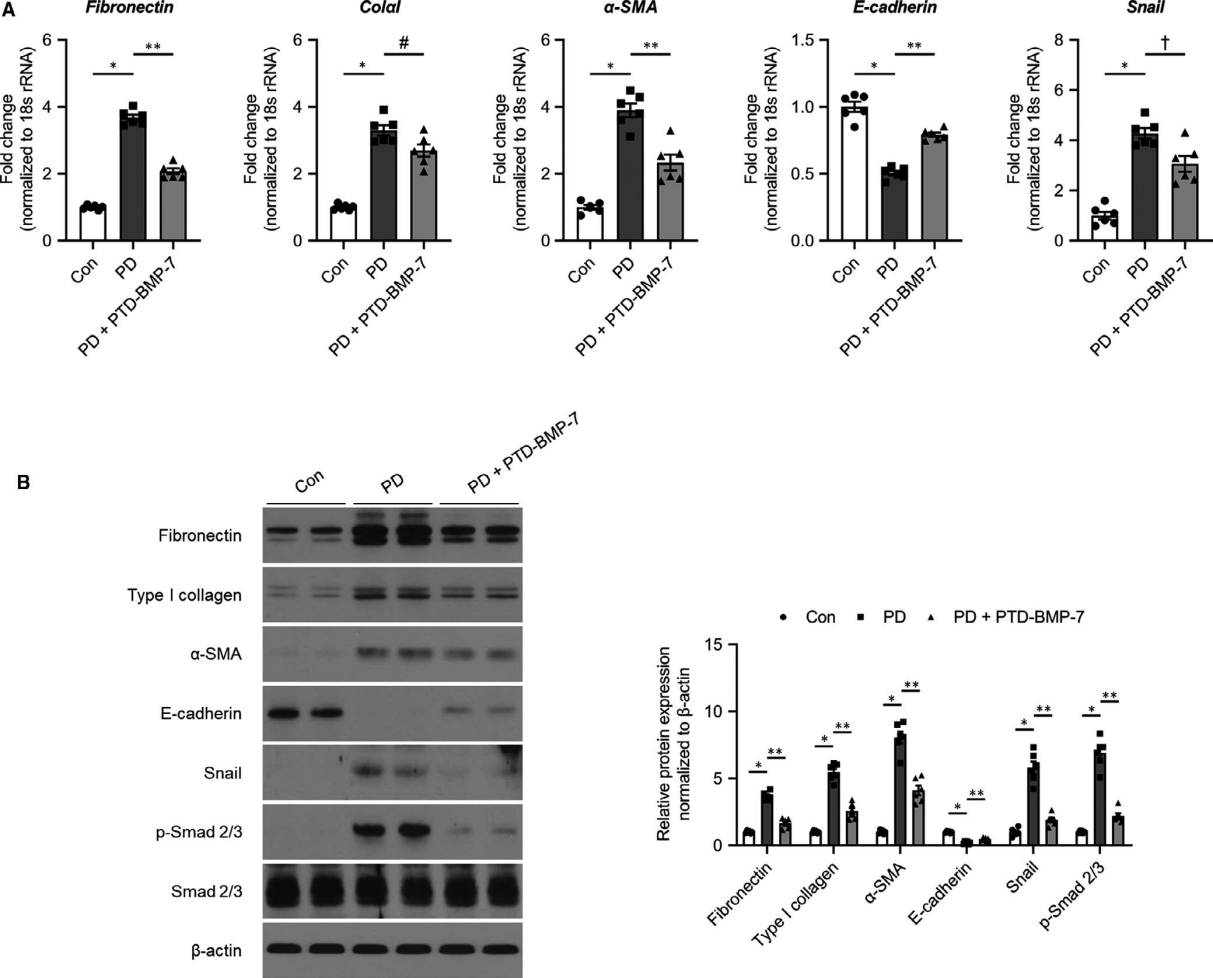
Effects of PTD‐BMP‐7 on peritoneal EMT‐related fibrosis in PD rat models. A, The mRNA expression of E‐cadherin was significantly lower, whereas the mRNA expressions of fibronectin, ColαI, α‐SMA and Snail were significantly higher in rats treated with PDF when compared to control rats. These changes were significantly abrogated by PTD‐BMP‐7. *P < .001 vs Con; **P < .001 vs PD; #*P *< .05 vs PD; †*P *< .01 vs PD. B, The protein levels of fibronectin, type I collagen, α‐SMA, E‐cadherin and Snail showed similar expression patterns to the mRNA expression in rats treated with PDF. Phospho‐Smad 2/3 protein expression, which induced EMT, was also significantly higher in rats treated with PDF. However, these changes in rats treated with PDF were significantly abrogated by PTD‐BMP‐7. *P < .001 vs Con; **P < .001 vs PD. n = 6 per group. Data were presented as mean ± SE. Con, control; PDF, 4.25% peritoneal dialysis fluid

**Figure 5:**
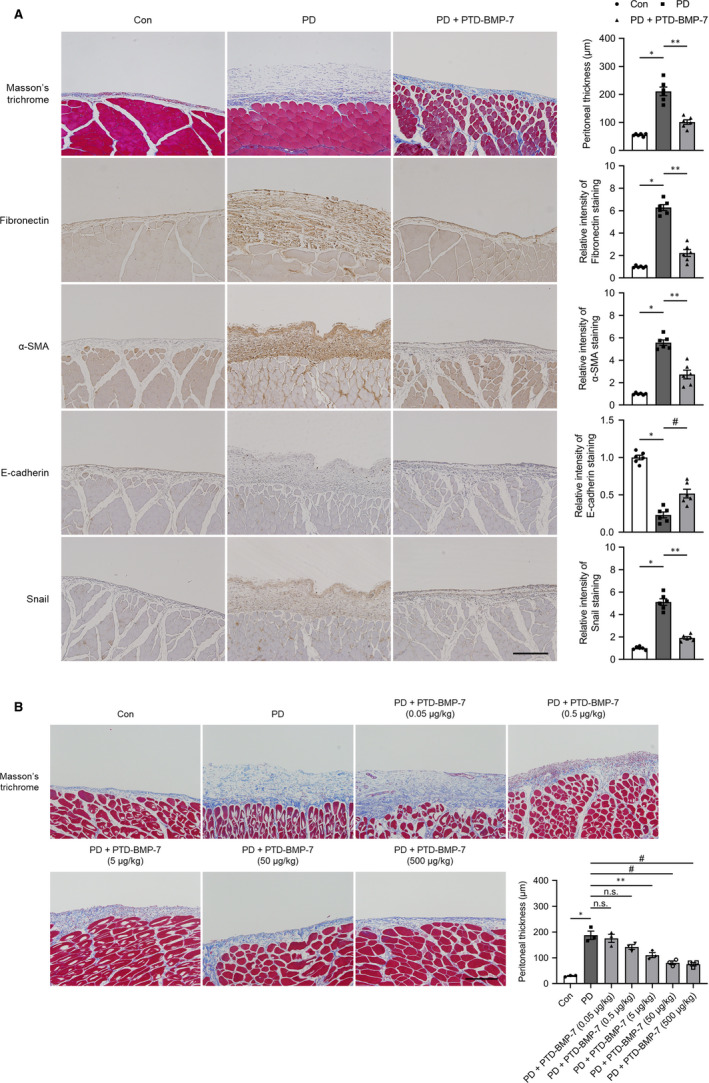
Masson's trichrome staining and immunohistochemical staining of the peritoneum in PD rat models. A, Peritoneal fibrosis assessed via Masson's trichrome staining was found to be significantly more extensive in PD rats than in control rats, and these changes were significantly attenuated by PTD‐BMP‐7. The intensity of E‐cadherin staining was significantly lower, whereas fibronectin, α‐SMA and Snail staining intensities were significantly higher in PD rats when compared to control rats. PTD‐BMP‐7 significantly ameliorated these changes in PD rats (×100). *P < .001 vs Con; **P < .001 vs PD; #*P *< .01 vs PD. B, PTD‐BMP‐7 treatment was effective for prevention of PDF‐induced peritoneal fibrosis in a dose‐dependent manner in 4‐weeks PD rat models. *P < .001 vs Con; **P < .001 vs PD; #*P *< .01 vs PD. n = 6 per group. Data were presented as mean ± SE. Scale bar = 200 µm. Con, control; PDF, 4.25% peritoneal dialysis fluid

The effects of PTD‐BMP‐7 on peritoneal EMT‐related fibrosis were explored in PDF‐induced PD fibrosis rat models. The mRNA expression of E‐cadherin was significantly lower (*P* < .001), whereas the mRNA expressions of fibronectin, ColαI, α‐SMA and Snail were significantly higher in rats treated with PDF compared to control rats (*P* < .001). These changes were significantly abrogated by PTD‐BMP‐7 (Figure 4A). The protein expressions of E‐cadherin, fibronectin, type I collagen, α‐SMA and Snail showed similar patterns to the mRNA expression in rats treated with PDF. PTD‐BMP‐7 normalized the increase in PDF‐induced EMT‐related protein expressions (Figure 4B). Furthermore, p‐Smad 2/3 protein expression was also significantly higher in PD rats compared to that of control rats. However, the p‐Smad 2/3 protein expression in PD rats was ameliorated by PTD‐BMP‐7 treatment (Figure 4B). Immunohistochemical analysis of the peritoneum also revealed that the expressions of α‐SMA, Snail and fibronectin were significantly higher in rats treated with PDF compared to control rats, and PTD‐BMP‐7 significantly abrogated the PDF‐induced fibrosis in PD rats (Figure 5A). In addition, Masson's trichrome staining demonstrated that the sub‐mesothelial layer and peritoneal fibrosis were significantly more increased in PD rats treated with PDF compared to control rats. These changes were significantly improved by PTD‐BMP‐7 treatment (Figure 5A). In addition, we found that PTD‐BMP‐7 treatment was effective for prevention of PDF‐induced peritoneal fibrosis in a dose‐dependent manner (Figure 5B).

### 3.4 Effects of PTD‐BMP‐7 on established peritoneal fibrosis

**Figure 6:**
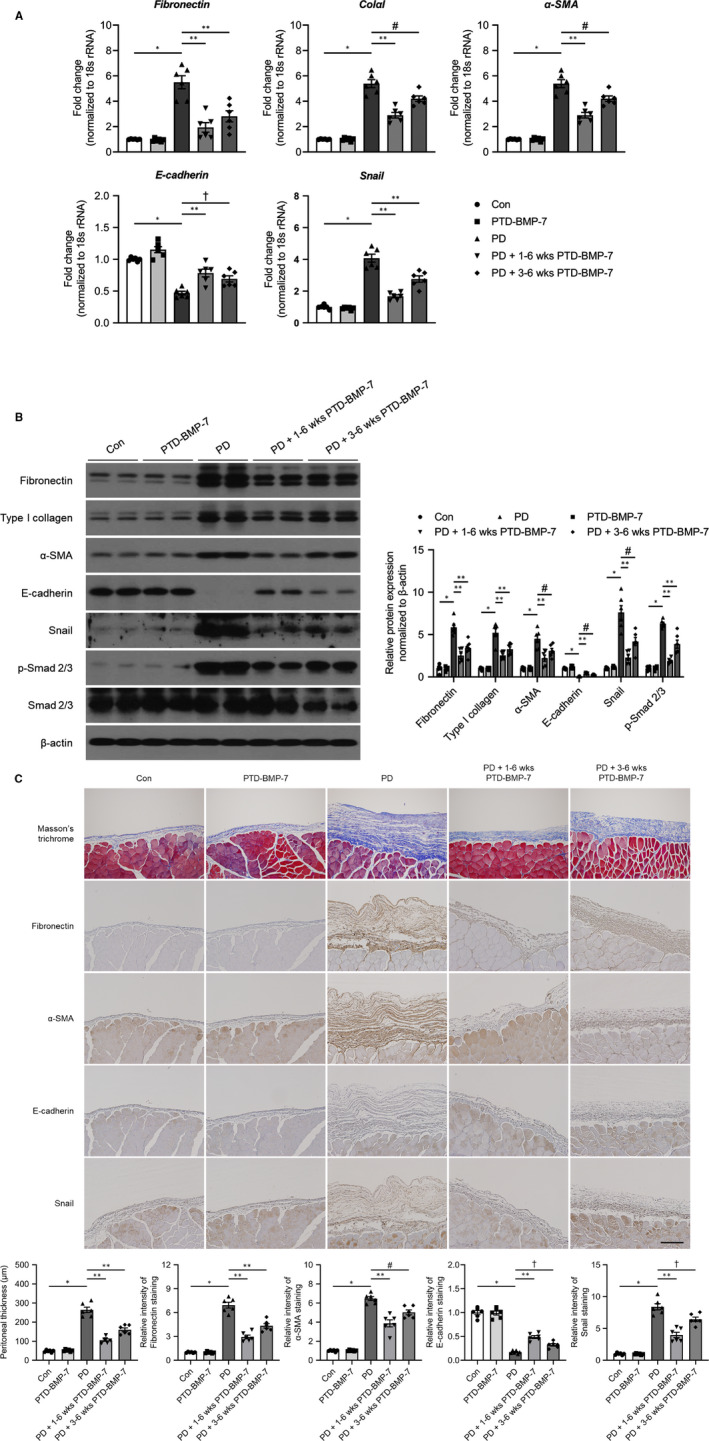
Effects of PTD‐BMP‐7 on established peritoneal fibrosis in 6‐wk PD rat models. A, The mRNA expression of E‐cadherin was significantly lower, whereas the mRNA expressions of fibronectin, ColαI, α‐SMA and Snail were significantly higher in rats treated with PDF when compared to control rats. These changes were significantly abrogated by PTD‐BMP‐7 administered 2 wk later. *P < .001 vs Con; **P < .001 vs PD; #*P *< .01 vs PD; †*P *< .05 vs PD. B, The protein levels of fibronectin, type I collagen, α‐SMA, E‐cadherin and Snail showed similar expression patterns to the mRNA expression. Phospho‐Smad 2/3 protein expression, which induced EMT, was also significantly higher in rats treated with PDF. However, the changes in rats treated with PDF were significantly abrogated by PTD‐BMP‐7 administered 2 wk later. *P < .001 vs Con; **P < .001 vs PD; #*P *< .05 vs PD. n = 6 per group. Data were presented as mean ± SE. Con, control; PDF, 4.25% peritoneal dialysis fluid. C, Peritoneal fibrosis assessed via Masson's trichrome staining was found to be significantly more extensive in PD rats treated with 4.25% PDF than in control rats. These changes were significantly attenuated by PTD‐BMP‐7 administered 2 wk later. The intensity of E‐cadherin staining was significantly lower, whereas fibronectin, α‐SMA and Snail staining intensities were significantly higher in PD rats when compared to control rats. PTD‐BMP‐7, administered 2 wk later, significantly ameliorated these changes in PD rats (×100). *P < .001 vs Con; **P < .001 vs PD; #*P *< .05 vs PD; †*P *< .01 vs PD. n = 6 per group. Data were presented as mean ± SE. Scale bar = 200 µm. Con, control; PDF, 4.25% peritoneal dialysis fluid

Next, we evaluated the effects of PTD‐BMP‐7 on established peritoneal fibrosis in 6‐week PD rat models. After peritoneal fibrosis had developed by treating PDF during 2 weeks period, PTD‐BMP‐7 was treated for subsequent 4 weeks. The mRNA expression of E‐cadherin was significantly lower, whereas mRNA expressions of fibronectin, ColαI, α‐SMA, and Snail were significantly higher in rats treated with PDF for 6 weeks when compared to control rats (*P* < .001). These changes were significantly abrogated even after delayed administration of PTD‐BMP‐7 (Figure 6A). The protein expressions related to EMT and fibrosis, that is E‐cadherin, fibronectin, type I collagen, α‐SMA and Snail were activated in 6‐week PD rat models, which were improved after PTD‐BMP‐7 administration. These EMT and fibrosis induced by PDF were partially recovered in rats subjected to the delayed PTD‐BMP‐7 treatment (Figure 6B). Immunohistochemical staining of fibronectin and Masson's trichrome staining also showed protective and improving anti‐fibrotic effect of PTD‐BMP‐7 in 6‐week PD rat models (Figure 6C). Of note, to evaluate the effects of PTD‐BMP‐7 on progressively established peritoneal fibrosis, PTD‐BMP‐7 was treated after 2 weeks of PDF treatment in 6‐week PD rat models. The mRNA and protein expression of E‐cadherin were significantly lower, whereas mRNA and protein expressions of fibronectin, type I collagen, α‐SMA and Snail were significantly higher in rats treated with PDF for 6 weeks when compared to control rats. These changes were significantly abrogated by PTD‐BMP‐7 administered after 2 weeks of PDF treatment. (Figure 6A,B). Masson's trichrome staining also showed anti‐fibrotic effect of PTD‐BMP‐7 on progressively established peritoneal fibrosis in 6‐week PD rat models (Figure 6C).

### 3.5 Comparison of the anti‐fibrotic effects of rBMP‐7 and PTD‐BMP‐7

**Figure 7:**
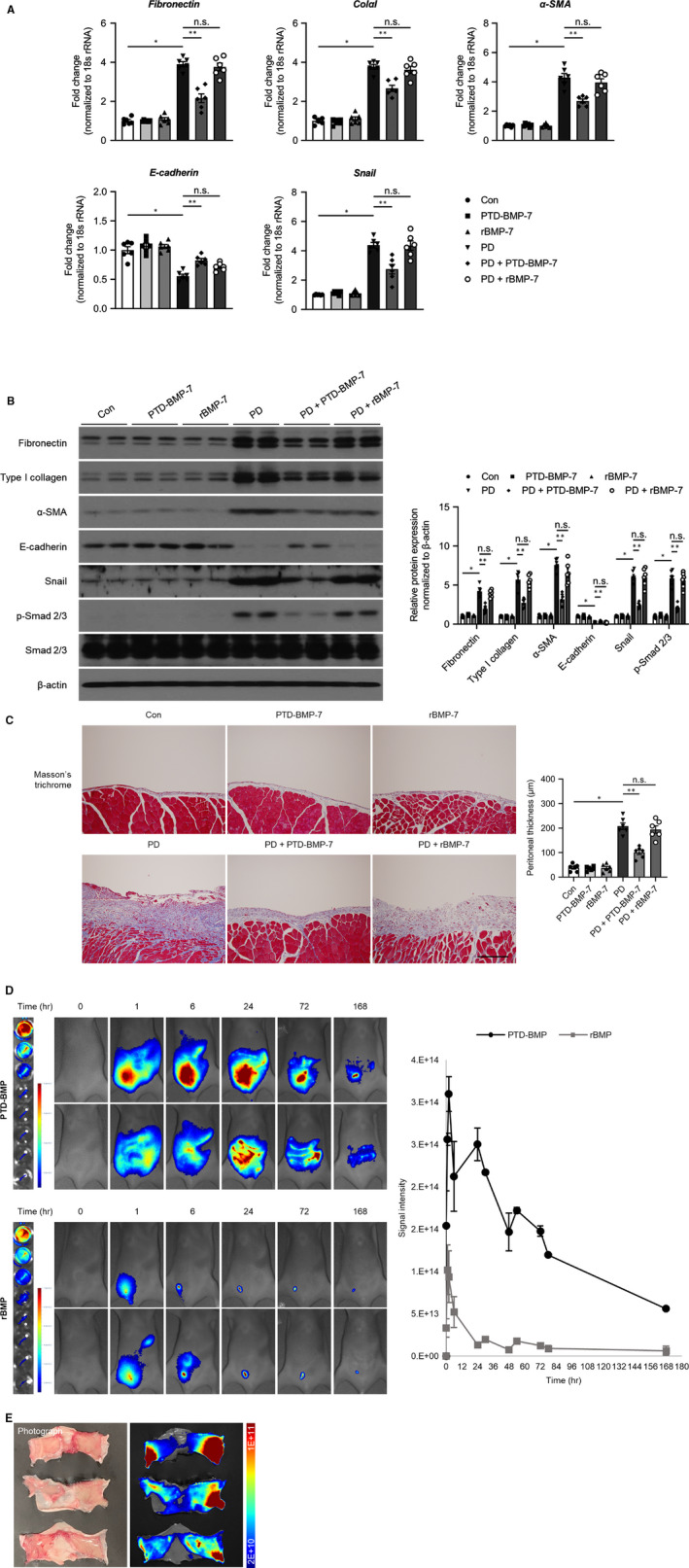
Comparison of the effects of rBMP‐7 and PTD‐BMP‐7. A, The mRNA expression of E‐cadherin was significantly lower, whereas the mRNA expressions of fibronectin, ColαI, α‐SMA and Snail were significantly higher in rats treated with PDF when compared to control rats. These changes were significantly abrogated only after administration of PTD‐BMP‐7. *P < .001 vs Con; **P < .001 vs PD. B, The protein levels of fibronectin, type I collagen, α‐SMA, E‐cadherin and Snail showed similar expression patterns to the mRNA expression in rats treated with PDF. Phospho‐Smad 2/3 protein expression, which induced EMT, was also significantly higher in rats treated with PDF. However, these changes in rats treated with PDF were significantly abrogated by only PTD‐BMP‐7. *P < .001 vs Con; **P < .001 vs PD. n = 6 per group. Data were presented as mean ± SE. Con, control; PDF, 4.25% peritoneal dialysis fluid. C, Peritoneal fibrosis assessed via Masson's trichrome staining was found to be significantly more extensive in PD rats treated with 4.25% PDF than in control rats. These changes were significantly attenuated by only PTD‐BMP‐7 administered. D, Monitoring by intravital in vivo fluorescence imaging, results of biodistribution showed that PTD‐BMP‐7 was more stably sustained than rBMP‐7. About 5 times longer half‐life was shown at PTD‐BMP‐7 (56.439 h) than rBMP‐7 (10.495 h), and about 9 time more area under the curve was shown at PTD‐BMP‐7 (9.861) compared with rBMP‐7 (1.000) in Balb/c nude mice. E, Monitoring by ex vivo fluorescence imaging, after 6 h from intra‐peritoneal injection of the ICG labelled PTD‐BMP, ICG signal was detected at skin and peritoneal membrane tissue on ex‐vivo fluorescence imaging. 18.5 (±8.289) % was delivered. Total signal intensity ({[p/s/cm^2^/sr]/[µW/cm^2^]} × cm^2^) of every time point was normalized by subtraction of background signal at 0 h (before injection). *P < .001 vs Con; **P < .001 vs PD. n = 6 per group. Data were presented as mean ± SE. Scale bar = 200 µm. Con, control; PDF, 4.25% peritoneal dialysis fluid

To confirm the effectiveness of PTD‐BMP‐7, the effects of rBMP‐7 and PTD‐BMP‐7 on peritoneal EMT‐related fibrosis were compared by same dosage. The mRNA and protein expression of E‐cadherin were significantly lower, whereas the mRNA and protein expressions of fibronectin, type I collagen, α‐SMA and Snail were significantly higher in rats treated with PDF when compared to control rats. These changes were significantly abrogated after administration of PTD‐BMP‐7, however, weekly administration of rBMP‐7 did not improve the PDF‐induced EMT‐related peritoneal fibrosis (Figure 7A,B). Masson's trichrome staining also showed that only PTD‐BMP‐7 had anti‐fibrotic effect in PD rat models (Figure 7C). In addition, to evaluate a progressive release of PTD‐BMP7, in vivo and ex vivo experiments were conducted. Compared to rBMP‐7, PTD‐BMP‐7 showed longer half‐life and bigger area under the curve which was calculated over the 1 week from the initial injection (Figure 7D). Of note, PTD‐BMP‐7 that was absorbed and maintained on the peritoneum membraned could be observed obviously. (Figure 7E).

## 4 DISCUSSION

In the present study, PTD‐BMP‐7, an indirect prodrug delivery system of BMP‐7, was shown to effectively block peritoneal membrane damage in in vitro and in vivo PD models. PTD‐BMP‐7 also partially delayed the progression of established PDF‐induced peritoneal fibrosis.

Long‐term exposure of the peritoneal membrane to glucose and non‐physiologic PDF induces peritoneal inflammation, thereby undergoing peritoneal fibrosis, which eventually leads to ultrafiltration failure.^23^ Although resident stromal fibroblasts have been recognized as the main origin of the myofibroblasts that cause these peritoneal membrane changes,^24^ recent studies have shown PMC EMT to also play an important role in these changes.^23^, ^25^, ^26^ EMT is a complicated process that includes the loss of cell polarity, loss of cell‐cell adhesion and acquisition of migration and invasive properties. This process induces PMCs to become myofibroblast‐like cells.^27^ Thus, PMCs that have undergone EMT invade the sub‐epithelial stroma and produce extracellular matrix (ECM) components, such as fibronectin and ColαI, which finally lead to peritoneal membrane fibrosis.^2^ It should be noted that a hyperglycaemic condition stabilizes and activates Snail via *O*‐GlcNAc, resulting in EMT.^28^

In the present study, TGF‐β1 treatment significantly decreased E‐cadherin expression and clearly increased α‐SMA and Snail expressions, accompanied by a significant increase in fibronectin expression in cultured HPMCs. A similar finding was also found in the peritoneal tissues of PD rats. The profibrotic cytokine, TGF‐β1, plays major roles to induce peritoneal EMT and fibrosis in patients undergoing PD.^29^ TGF‐β1 activates downstream Smad 2/3 cascades, which consequently results in molecular transition, down‐regulating intracellular adhesion molecules such as E‐cadherin and ZO‐1 and up‐regulating mesenchymal molecules such as α‐SMA, Snail and fibronectin.^2^, ^29^ However, the effect of TGF‐β1 on various physiologic functions related to cell survival, should not be overlooked.^30^ Attempting to inhibit TGF‐β1 for preventing organ fibrosis arising from chronic state of various disease^31^ may block numerous critical downstream signalling pathways in regard to cell survival.^29^ Therefore, the clinical use of TGF‐β1‐inhibiting therapy has been limited. On the other hand, BMP‐7 is an endogenous protein that antagonizes TGF‐β1 and reverses organ fibrosis caused by chronic state of various disease.^32^, ^33^, ^34^, ^35^ In controlling EMT and fibrosis, a delicate balance between TGF‐β1 and BMP‐7 is maintained.^36^ They bind to specific type I serine‐threonine kinase receptors and induce distinct intracellular signals via the Smad‐dependent pathway.^7^, ^8^, ^9^ Particularly, BMP‐7 inhibits the activation of both, the Smad 2/3‐dependent and Smad‐independent pathways, via the activation of Smad 1/5/8.^37^, ^38^, ^39^ Interestingly, Lourerio et al showed that exogenous rhBMP‐7 completely inhibits the TGF‐β1‐induced EMT of HPMCs in vitro and ameliorates peritoneal fibrosis in PD rat models.^3^ However, it is difficult to apply exogenous rhBMP‐7 into clinical practices because of the short biological half‐life of rhBMP, brought about by fast enzymatic degradation.^10^, ^11^ Furthermore, soluble rhBMP‐7 cannot be applied in hypertonic conditions because the hydrogen bond in the tertiary structures of growth factors is easily destroyed by pH or osmotic pressure.^40^ By solving the structural problems of recombinant proteins, they can be expected to demonstrate clinical value as therapeutic agents. The present study suggests that binding PTD without specific carriers, like those in viral transfections, increases efficiency and overcomes structural problems of rhBMP‐7.

The eukaryotic cell membrane consists of a lipid bilayer with embedded proteins. Normally, most peptides and proteins cannot across the cell membrane because of the hydrophobicity of these lipids. However, the discovery of several PTDs, also known as cell‐penetration proteins (CPPs) made an interesting exception to this rule.^41^ Until now, various types of CPPs have been identified such as Antennapedia (Antp), herpes simplex virus type 1 (HSV‐1) protein VP22, HIV‐derived cationic TAT, transportan and polyarginine sequences (Arg8).^42^ TAT peptides, in particular, are predominantly a type of CPP, with a distinguished ability to across the eukaryotic cell membrane, that are used to deliver peptides and proteins into cells.^43^ This ability of the TAT peptides to be used as intracellular delivery of large molecules has received much interest since Frankel and Pabo ^13^ discovered that HIV‐derived cationic TAT could enter cells. The first proof‐of‐concept of the in vivo application of PTD in delivering small peptides and large proteins was reported by Dowdy's group.^17^ Protein transduction involves a three‐step process: (a) binding of PTD to the cellular membrane, (b) cellular uptake by endocytosis and (c) endosome‐to‐cytosol transport. Although the precise mechanism of endosome‐to‐cytosol transport is not clear yet, pH drop in maturing endosomes is thought to be crucial elements.^14^

Recently, we developed a PTD‐based rBMP‐2 polypeptide (PTD‐BMP‐2) to delivery polypeptides into cells, followed by furin‐mediated protein cleavage and secretion of active growth factors from the cells.^12^ The study demonstrated an indirect delivery system using a PTD‐protein, thereby mimicking endogenous protein biogenesis. As a denatured polypeptide, the prodrug was also resistant to proteolytic degradation via body fluids. In fact, PTD‐BMP‐2 was functional during in vivo bone regeneration, with only a few micrograms being sufficient for the process.^12^ In line with these findings, the PTD‐BMP‐7 dosage (10 μg/wk) instilled intra‐peritoneally in the present study was significantly smaller when compared to the rBMP‐7 dosage (100‐300 μg/kg/d) used to treat peritoneal fibrosis in PD rat models in other studies.^3^, ^32^ An EMT‐suppressing effect was observed with the PTD‐BMP‐7 dose used in this study. Moreover, we also showed that early or late PTD‐BMP‐7 treatments reduced EMT and confirmed the effect of PTD‐BMP‐7 on established peritoneal fibrosis. Therefore, the results of the present study suggested that PTD‐BMP‐7 effectively blocked peritoneal membrane damage in PD rat models.

In summary, PTD‐BMP‐7 significantly blocked TGF‐β1‐induced EMT of HPMCs. Moreover, low PTD‐BMP‐7 doses significantly ameliorated this PD‐induced peritoneal fibrosis in animal models. These findings suggest that PTD‐BMP‐7 treatment is considered to be an effective treatment modality for peritoneal fibrosis in PD patients.

## CONFLICT OF INTEREST

J.I.Y. is an inventor of the patent related to this work filed by MET Life Sciences., Ltd. (Korean Patent Application Number: 10‐2019‐0076443, PCT Application Number: PCT/KR2020/007011). N.H.K., H.S.K., T.‐H.Y., and J.I.Y. are the founders of MET Life Sciences Co., Ltd. and shareholder. All other authors declare that they have no competing interests.

## AUTHOR CONTRIBUTIONS

**Seonghun Kim:** Data curation (equal); formal analysis (equal). **Dong Ho Shin:** Conceptualization (equal); writing‐original draft (equal). **Bo Young Nam:** Data curation (equal); methodology (equal). **Hye‐Young Kang:** Supervision (equal). **Jimin Park:** Methodology (equal); validation (equal). **Meiyan Wu:** Investigation (equal); visualization (equal). **Nam Hee Kim:** Supervision (equal). **Hyun Sil Kim:** Supervision (equal). **Jung Tak Park:** Supervision (equal). **Seung Hyeok Han:** Supervision (equal). **Shin‐Wook Kang:** Validation (equal). **Tae‐Hyun Yoo:** Conceptualization (equal); project administration (equal); supervision (equal); writing‐review and editing (equal). **Jong In Yook:** Conceptualization (equal).

## Supporting information

Table S1

## Data Availability

The data that support the findings of this study are available from the corresponding author upon reasonable request.
