# Supplementary material for: Newly designed Protein Transduction Domain (PTD)‐mediated BMP‐7 is a potential therapeutic for peritoneal fibrosis
Source: J Cell Mol Med. 2020 Oct 20;24(22):13507–22. doi: 10.1111/jcmm.15992 (PMC7701504; doi:10.1111/jcmm.15992)
Supplement: Supplementary file 1 — Table S1 [file JCMM-24-13507-s001.docx]

|  | Sequence | |
| --- | --- | --- |
| ***Fibronectin*** | Sense | 5’-AACCTACGGATGACTCGTGC-3’ |
| (human) | Antisense | 5’-TGAATCACATCTGAAATGACCAC-3’ |
| ***Fibronectin*** | Sense | 5’-GCGACTCTGACTGGCCTTAC-3’ |
| (rat) | Antisense | 5’-CCGTGTAAGGGTCAAAGCAT-3’ |
| ***Collagen type I (Col1a1)*** | Sense | 5’-CCAAATCTGTCTCCCCAGAA -3’ |
| (human) | Antisense | 5’-TCAAAAACGAAGGGGAGATG-3’ |
| ***Collagen type I (Col1a1)*** | Sense | 5’-AGG AGA GAG TGC CAA CTC CA-3’ |
| (rat) | Antisense | 5’-CCA CCC CAG GGA TAA AAA CT-3’ |
| ***α-SMA*** | Sense | 5’-GCCTTGGTGTGTGACAATGG-3’ |
| (human) | Antisense | 5’-AAAACAGCCCTGGGAGCAT-3’ |
| ***α-SMA*** | Sense | 5’-TTCAATGTCCCTGCCATGTA-3’ |
| (rat) | Antisense | 5’-GAAGGAATAGCCACGCTCAG-3’ |
| ***E-cadherin*** | Sense | 5’-CACAGACGCGGACGATGAT-3’ |
| (human) | Antisense | 5’-AGGATCTTGGCTGAGGATGGT-3’ |
| ***E-cadherin*** | Sense | 5’-GGG TTGTCTCAGCCAATGTT-3’ |
| (rat) | Antisense | 5’-CACCAACACACCCAGCATAG-3’ |
| ***Snail*** | Sense | 5’-ATCCTCACCTCGGGAGCATAC-3’ |
| (human) | Antisense | 5’-AGGCCACTGGGTAAAGGAGAGT-3’ |
| ***Snail*** | Sense | 5’-TGAGAAGCCTTTCTCCTGCT-3’ |
| (rat) | Antisense | 5’-CTGGTATCTCTTCACATCCG-3’ |
| ***18s*** | Sense | 5’-TGGTGCATGGCCGTTCT-3’ |
| (human) | Antisense | 5’-CATGCCAGAGTCTCGTTCGTT-3’ |
| ***18s***  (mouse) | Sense | 5’-CATGTCTAAGTACGCACGGC-3’ |
| (rat) | Antisense | 5’-CAAGTAGGAGAGGAGCGAGC-3’ |

**Supplementary Table 1. Primer sequences**
